# A Method for Improving the Detection Accuracy of the Spot Position of the Four-Quadrant Detector in a Free Space Optical Communication System

**DOI:** 10.3390/s20247164

**Published:** 2020-12-14

**Authors:** Xuan Wang, Xiuqin Su, Guizhong Liu, Junfeng Han, Kaidi Wang, Wenhua Zhu

**Affiliations:** 1 Xi’an Institute of Optics and Precision Mechanics, Chinese Academy of Sciences, Xi’an 710119, China; suxiuqin@opt.ac.cn (X.S.); hanjf@opt.ac.cn (J.H.); wangkaidi@opt.cn (K.W.); zhuwenhua2015@opt.cn (W.Z.); 2 School of Electronic and Information Engineering, Xi’an Jiaotong University, Xi’an 710049, China; liugz@mail.xjtu.edu.cn; 3 University of Chinese Academy of Sciences, Beijing 100049, China

**Keywords:** quadrant detectors, Gaussian spot, high background noise

## Abstract

In a free space optical communication system, the beacon light will lose most of its energy after long-distance transmission, and the background light from the universe will strongly interfere with it. The four-quadrant detector (4QD) has been widely used in optical communication systems as a high-precision spot position detection sensor. However, if the light signal falling on the 4QD is too weak, the electrical signal of the output position will be very weak, and it will easily be affected by or even submerged in noise. To solve this problem, we propose a method for improving the spot position detection accuracy. First, we analyzed the solution relationship between the actual position of the spot and the output signal of the 4QD, with a Gaussian spot as the incident light model. The output current signal of the detector was then transimpedance-amplified by an analog circuit and the output voltage signal with noise was digitally filtered. An error compensation factor and the gap size of the detector were introduced into the traditional spot position detection model. High-precision spot position information for the 4QD in a complex environment was then obtained using the improved spot position detection model. Experimental results show that the maximum spot position detection error for this method was only 0.0277 mm, and the root mean square error was 0.0065 mm, when the 4QD was in a high background noise environment. The spot position detection accuracy was significantly improved compared with traditional detection algorithms. Real-time detection can therefore be achieved in practical applications.

## 1. Introduction

Compared with a traditional microwave communication system, a free space optical communication system has more favorable transmission characteristics. It has higher bandwidth, lighter system weight, higher confidentiality, and lower power consumption requirements [1,2]. In order to achieve high speed, large capacity, high spectral efficiency, and long-distance transmission, space optical communication systems are developing rapidly. Researchers have done a lot of research on improving system performance, including improving communication technology to improve system performance [3,4], improving the stability of optical communication links during transmission and increasing optical communication transmission efficiency. The pointing, acquisition, and tracking (PAT) of the beam as the fundamental guarantee for link maintenance in the free space optical communication system also faces more challenges.

This is because the optical system is extremely susceptible to dynamic interference from the environment after long-distance transmission. For example, temperature changes in the environment, atmospheric turbulence, dust, smoke, and various mechanical vibrations will affect the performance of the optical system to varying degrees. Moreover, in the free space optical communication system, because the divergence angle of laser beam is small and the receiving field of view is narrow, the communication party must maintain precise aiming and tracking [5,6,7,8]. The key technology that determines the long-distance laser beam stability control and tracking in the free space optical communication system is the high-precision detection of the laser spot position [9].

The four-quadrant detector (4QD) is a key component in a beam stabilization system. Its main advantages are its high position resolution, simple processing circuit and high position detection accuracy. The spot position detection accuracy determines the control accuracy of the system [10,11]. However, the 4QD is very sensitive to ambient light, and the influence of ambient light such as sunlight on the detection accuracy cannot be ignored in practical use. In addition, the relationship between the output signal of the 4QD and the true position of the light spot on the photosensitive surface is complicated, and there is an inevitable gap between each quadrant on the photosensitive surface of the detector. Therefore, some researchers have developed detection algorithms that can improve the spot position detection accuracy of the 4QD [12,13,14,15,16].

Mengwei Chen et al. expanded the 4QD measurement range by changing spot energy distribution and spot shape [17]. Song-Cu et al. proposed a new spot position calculation algorithm, which improves the accuracy of 4QD spot position detection, and also improves the detection range under different spot sizes [18,19]. Wu Jiabin et al. proposed an innovative infinite integral model to reduce the position detection errors for different radii [20]. Qing Li et al. analyzed the influence of signal-to-noise ratio on 4QD detection accuracy and used the cyclic cross-correlation method to denoise the modulated light to improve detection accuracy [21]. The aforementioned methods are used to calculate the spot position directly through the 4QD output photocurrent. When the photoelectric signal contains significant noise signals, the detection accuracy will be affected non-negligibly. In addition, we need to consider not only denoising processing, but also the elimination of errors caused by the spot size and the blind area of the photosensitive surface in the spot position calculation algorithm.

In this paper we propose a method with higher position detection accuracy for spot position detection under the influence of complex ambient light, in which the low signal-to-noise ratio (SNR) spot position signal is filtered and denoised by digital signal filtering. Based on the new position detection model, the position detection accuracy and linear measurement range of the 4QD under low SNR are effectively improved. First, we analyze and compared the degree of influence of background light on 4QD detection accuracy, and analyze the influence of noise on the detection accuracy of the spot position by establishing a mathematical model. Combined with the previous work, we then analyze the influence of the 4QD itself on the spot position detection accuracy, and also determine the factors that will affect the spot position detection error. We introduce an error compensation factor containing information regarding the detector gap size and the spot radius into the preliminary solution value, and then obtain an improved Gaussian spot position detection algorithm by polynomial fitting. The experimental results show that the new detection model effectively improves the position detection accuracy and greatly improves the detection linearity of the detector in a large range. Therefore, this method has potential application value for practical use with ambient light interference.

This paper is organized as follows: In Section 2, the principle of 4QD measurement and the main factors affecting its accuracy are introduced. In Section 3, the method for improving the accuracy of spot position detection in a high-background-noise environment is introduced in detail. Section 4 introduces the spot position detection experiment based on the 4QD. Section 5 summarizes our conclusions.

## 2. System Description and Operating Principles of the 4QD

A free space optical communication system has more powerful functions than a traditional radio communication system. It has a higher information transmission bandwidth, stronger confidentiality, a smaller divergence angle and a larger transmission distance than traditional radio frequency systems. However, there are challenges involved in the practical use of a free space optical communication system. One such challenge is that the long-distance transmission of a laser beam is susceptible to the jitter caused by platform vibrations and atmospheric turbulence. Therefore, a 4QD can be used in the system to detect the spot position error and feedback to the beam correction mechanism to suppress the beam jitter.

**Figure 1:**
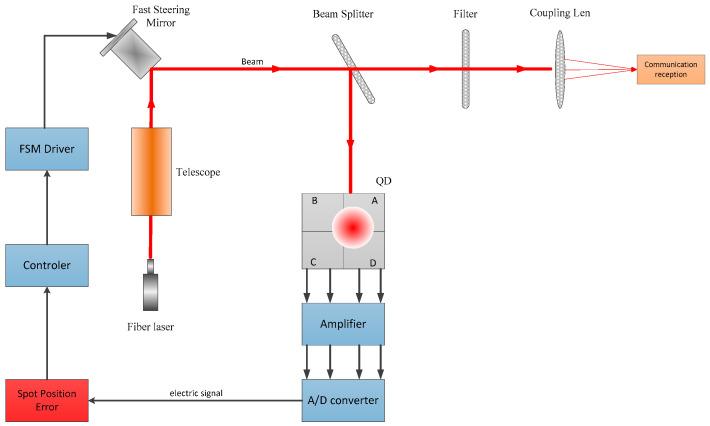
Block diagram of free space optical communication system.

A block diagram of the free space optical communication beam stabilization control system is shown in Figure 1. The 4QD consists of four identical InGaAs photodiodes. When the 4QD detects the beacon light signal, it will feedback the spot error information to the controller, which then controls the fast steering mirror (FSM) to correct the beam jitter. Through this closed-loop control, high-precision beam pointing, aiming, and tracking control can be achieved.

**Figure 2:**
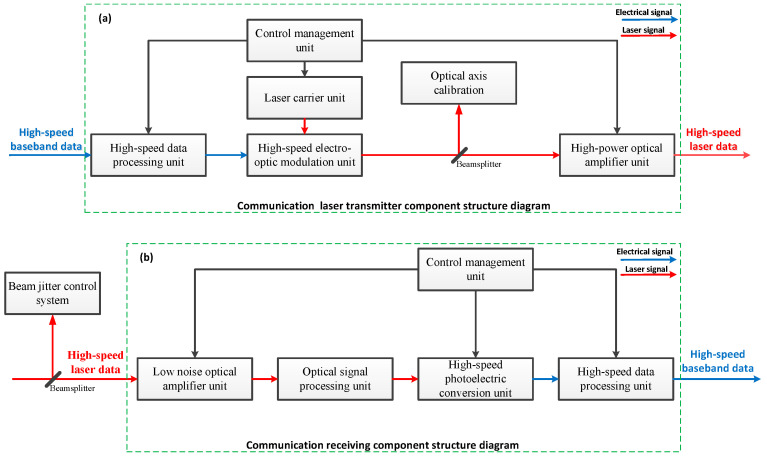
(**a**) Principal block diagram of communication laser transmitter component; (**b**) principal block diagram of communication laser receiver component.

In the free space optical communication system, the communication light emitted by the laser is completed by the optical communication transmitter module, and the electric signal that needs to be transmitted is loaded on the communication light. After long-distance transmission is received by the communication receiving module, the communication receiving module demodulates the electrical signal from the received optical signal. The stability of the communication optical link is guaranteed by the beam stability control system. The communication element of the free space optical communication system comprises a communication transmitter module and a communication receiving module. System structure diagrams of the communication transmitter and receiver modules are shown in Figure 2.

**Figure 3:**
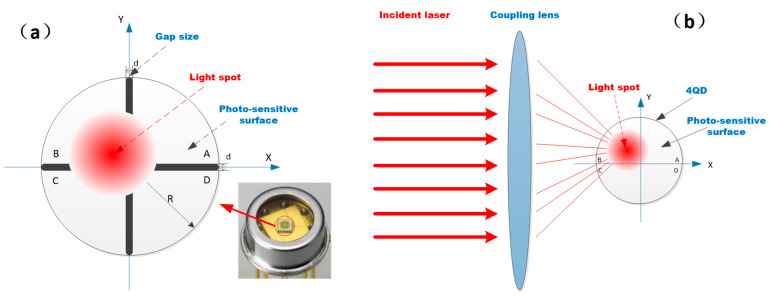
(**a**) Schematic diagram of the four-quadrant detector structure; (**b**) principles of laser spot detection of quadrant detector.

The 4QD has been widely used in high-energy laser transmission, satellite-to-ground laser communication, laser processing and other fields. Figure 3 shows a photograph of the 4QD, as well as the structure of the photosensitive surface and the detection principles.

The photosensitive surface of the 4QD is divided into four pieces of equal size according to the Cartesian coordinate system; the four photosensitive surfaces are the four quadrants of the 4QD. When the light spot falls on the photosensitive surface, each quadrant generates a corresponding photocurrent, whose magnitude corresponds to the magnitude of the light energy incident on each quadrant. There is a gap between each quadrant, also called a dead zone, which cannot be avoided due to the manufacturing process. Researchers have proven that the size of this gap will affect the accuracy of spot position detection.

When the laser beam is focused on the detector surface through the coupling lens, the position coordinates of the center point of the laser spot are (*x*, *y*), the light energy distributed on each quadrant of the laser spot with Gaussian distribution is $P_{A}$, $P_{B}$, $P_{C}$, $P_{D}$ and the corresponding photocurrent is $I_{A}$, $I_{B}$, $I_{C}$, $I_{D}$. Therefore, the position of the center of the light spot on the photosensitive surface can be calculated by the magnitude of the photocurrent in each quadrant [22]. The coordinates of the spot center position can be expressed by Equations (1) and (2).

(1) $$E_{x}=\frac{\left(I_{A}+I_{D})-(I_{B}+I_{C}\right)}{I_{A}+I_{B}+I_{C}+I_{D}}=\frac{\left(P_{A}+P_{D})-(P_{B}+P_{C}\right)}{P_{A}+P_{B}+P_{C}+P_{D}},$$

(2) $$E_{y}=\frac{\left(I_{A}+I_{B})-(I_{C}+I_{D}\right)}{I_{A}+I_{B}+I_{C}+I_{D}}=\frac{\left(P_{A}+P_{B})-(P_{C}+P_{D}\right)}{P_{A}+P_{B}+P_{C}+P_{D}},$$

where $E_{x}$ and $E_{y}$ represent the estimated values of the spot position coordinates calculated from the four quadrants’ photocurrents. We can roughly estimate the position of the light spot using these two values. The error between this value and the actual position of the light spot is relatively large. Therefore, it is necessary to calculate the actual position of the light spot on the surface of the detector through a calculation algorithm.

In practical applications, because the 4QD is susceptible to the influence of light noise in the environment and dark current, it is necessary to include noise components in the detector output photocurrent [23,24]. Since the *x*- and *y*-axes of the detector are independent of each other, we can analyze the *x*-axis coordinates separately, and Equation (1) can be rewritten as

(3) $$E_{x}=\frac{\left(I_{A}+I_{nA}+I_{D}+I_{nD})-(I_{B}+I_{nB}+I_{C}+I_{nC}\right)}{I_{A}+I_{B}+I_{C}+I_{D}+I_{nA}+I_{nB}+I_{nC}+I_{nD}},$$

where $I_{nA}$, $I_{nB}$, $I_{nC}$, and $I_{nD}$ represent the noise terms for each quadrant, and noise terms include the background noise current and the dark current for each quadrant.

Therefore, the error of the Gaussian spot on the *x*-axis can be written as

(4) $$\Delta x=erf\left(\frac{\sqrt{2}x_{0}}{\omega }\right),$$

where $erf()=\frac{2}{\sqrt{\pi }}\int e^{-t^{2}}dt$ is the error function. Then, the variance of Δx can be written as

(5) $$\sigma_{\Delta x}^{2}=\frac{1+erf^{2}(\frac{\sqrt{2}x_{0}}{\omega })}{SNR},$$

From Equation (5), we can derive that increasing the SNR of the photoelectric signal is an effective means by which to improve the accuracy of spot position detection. However, the power of the beacon laser will be attenuated after being transmitted over a long distance. It may even be submerged in the background light noise. Therefore, the spot position accuracy obtained by the direct detection method will be reduced. In this paper, our new detection method is based on a denoising algorithm.

## 3. Method for Improving 4QD Detection Accuracy

In a practical environment, the primary factors that reduce the output SNR of the 4QD are the background light noise current and the dark current from the 4QD. We use two main methods to improve 4QD detection accuracy in complex environments. The core processing method involves 4QD output signal denoising. We use a low-pass filter circuit to filter out high-frequency noise in a transimpedance amplifier circuit, perform analog signal to digital signal(A/D) conversion on the amplified voltage signal and then perform digital filtering. Next, we use a beacon laser electrical signal that is closer to reality to calculate the spot position. This step is required to calculate the spot position through an improved spot position calculation algorithm.

### 3.1. Denoising in a High-Background-Noise Environment

**Figure 4:**
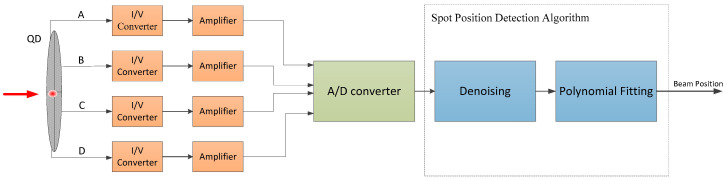
Photocurrent detection flow chart.

First, we consider the improvement of detection accuracy in a high-background-light environment. As described above, the photocurrent in each quadrant is amplified and converted from current to voltage through a transimpedance amplifier circuit, and then low-pass filtered in the analog amplifier circuit. However, this is not enough to effectively remove the background light noise and dark current noise in the 4QD output signal. The NI6115 acquisition card collects the converted voltage signal on the host computer, and we use a Kalman filter to filter the output signal of each quadrant of the 4QD. The collection and processing flow of the photocurrent signal is shown in Figure 4. Since the background light noise and dark current noise exhibit a Gaussian distribution [25], the Kalman filter algorithm processes the output signal and filters the noise more efficiently, so that the information regarding the center position of the laser spot is more accurate.

The Kalman filtering algorithm only needs to store the current state and parameters, that is, it can predict and analyze the next moment without occupying too many hardware resources to achieve a faster calculation speed. Therefore, before performing the calculation of the spot position, performing Kalman filtering on the output current of the 4QD can further improve the accuracy of the center position of the laser spot, thereby improving the tracking accuracy of the free space optical communication system. Under the influence of white noise, the linear discrete Kalman filter treats signal processing as the output of a linear system [26]. We use a state equation to describe this process.

The state equation is

(6) $$X(k)=F_{k,k-1}X(k-1)+\Gamma_{k-1}W(k-1),$$

(7) $$Z(k)=H_{k}X(k)+V(k),$$

where k represents the discrete time of the entire system and X(k) indicates the current state of the system. At this time, the system state is the magnitude of the photoelectric signal collected in each quadrant at the current moment. X(k−1) is the state of the system at time k−1, that is, the magnitude of the photoelectric signal at the previous time. Z(k) indicates the observation signal, that is, the photoelectric signal of each quadrant that was measured during the measurement process. W(k−1) represents the white noise of the input system, which is part of the process noise. V(k) is the observation noise, that is, the environmental noise and dark current noise that were detected in the photoelectric detection process. F is the state transition matrix, Γ is the noise driving matrix and H is the observation matrix.

The object of our research is the photoelectric signal output from the four quadrants of the 4QD. The magnitude of the photoelectric signal is related to the magnitude of the light energy received in each quadrant. We know in advance that the incident spot model is a Gaussian laser spot, and we can determine the theoretical photocurrent output value of each quadrant by establishing a spot position model. Due to the influence of noise on the output photocurrent, the magnitude of the output electrical signal in the four quadrants will change. In a short period of time, we believe that the current state is equal to the state of the previous moment. Our observation is the value of the state plus the observed noise. The state of the system and the observation equation can then be simplified.

(8) X(k)=X(k−1),

(9) Z(k)=X(k)+V(k),

In this experiment, the Kalman filter algorithm is gradually recursive according to the following process.

The one-step prediction equation of state is

(10) $${\hat{X}}_{k/k-1}=F_{k,k-1}X_{k-1},$$

The one-step prediction of mean square error is

(11) $${\hat{P}}_{k/k-1}=F_{k,k-1}P_{k-1}F_{k,k-1}^{T}+\Gamma_{k-1}Q_{k-1}\Gamma_{k-1}^{T},$$

where Q is the covariance of the process noise.

The Kalman gain equation is

(12) $$G_{k}={\hat{P}}_{k/k-1}H_{k}[H_{k}{\hat{P}}_{k/k-1}H_{k}^{T}+R_{k}{]}^{-1},$$

where R is the covariance of the measurement noise.

The filtering estimation equation is

(13) $$X_{k}={\hat{X}}_{k/k-1}+G_{k}[Z_{k}-H_{k}{\hat{X}}_{k/k-1}],$$

The mean square error update matrix is

(14) $$P_{k}=[I-G_{k}H_{k}]{\hat{P}}_{k/k-1},$$

The value obtained after Kalman filtering is closer to the true value than the measured value. Therefore, after Kalman filtering the output values of the four quadrants, we introduce them into the spot position calculation algorithm, which can effectively improve the spot position detection accuracy.

**Figure 5:**
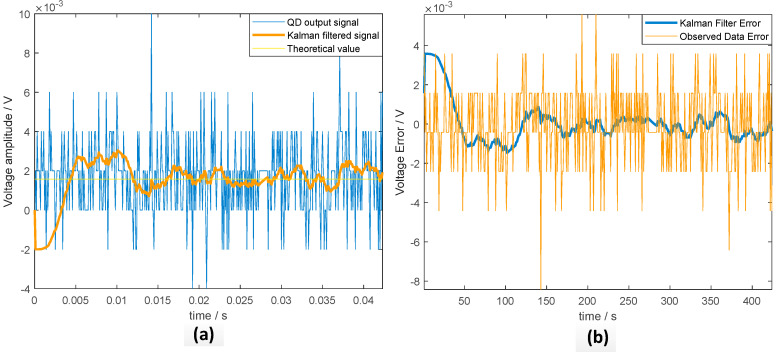
(**a**) Four-quadrant detector output signal Kalman filter result; (**b**) Kalman filter error.

We analyze the filtering effect by Kalman filtering the photocurrent collected in the experiment for one quadrant. It can be seen from Figure 5 that the background photocurrent noise and dark current noise have a significant influence on the 4QD output electrical signal, and the accuracy of the electrical signal after Kalman filter processing is greatly improved compared to the signal accuracy obtained by direct observation.

### 3.2. Improvement of Spot Position Detection Model

After obtaining the ideal photocurrent signal for each quadrant on the 4QD, we need to calculate the actual position of the spot on the surface of the detector through a solver algorithm. We have made some improvements to the traditional spot position calculation model. The improvement of the model can further improve the spot position detection accuracy and the detection linearity in a large range from the calculation method. First, we establish a new spot position detection model, which takes into account the influence of the detector gap size. Secondly, we introduce an error compensation term that includes the size of the spot in the new model [27].

After the laser is transformed by the optical system, it falls on the 4QD photosensitive surface and the spot energy distribution is usually a Gaussian dot. The expression of the spot energy of the Gaussian distribution is

(15) $$h(x,y)=\frac{2P_{0}}{\pi \omega^{2}}\exp\left[-\frac{2{\left({\left(x-X\right)}^{2}+(y-Y)\right)}^{2}}{\omega^{2}}\right],$$

where $p_{{}_{0}}$ is the total energy of the spot, ω is the beam waist radius of the Gaussian spot and (*X*, *Y*) is the coordinate position of the centroid of the spot.

Since the 4QD is formed by splicing four detectors, there will be a gap in the splicing area. Even if the size of the gap is small, the optical signal cannot generate photocurrent in this area. Therefore, when we consider the size of the gap in the model, the optical power of each quadrant can be expressed by the following equations

(16) $$P_{A}+P_{D}=\iint_{S_{A}+A_{D}}h(x,y)dxdy-\iint_{S_{gap}}h(x,y)dxdy,$$

(17) $$P_{B}+P_{C}=\iint_{S_{B}+A_{C}}h(x,y)dxdy-\iint_{S_{gap}}h(x,y)dxdy,$$

where $P_{A}$, $P_{B}$, $P_{C}$, $P_{D}$, represents the intensity of the light spot falling on each quadrant of the detector surface, $S_{A}$, $S_{B}$, $S_{C}$, $S_{D}$, represents the area size of the light spot falling on each quadrant of the detector surface, and $S_{gap}$ represents the size of the blind area of the light spot falling on the detector surface. In this way, we introduce the size of the detector blind zone into the model.

The spot position obtained by the direct method is only an approximate value, and because the accuracy is not high enough, we call it the preliminary solution value. Therefore, we improve the detection accuracy of the spot position on the detector by establishing a new detection model. With the introduction of the error compensation function $\lambda (x_{0})$, the preliminary solution value of the spot position can be rewritten as

(18) $$x=g(E_{x})\cdot \omega \cdot \lambda (x_{0}),$$

where $g(E_{x})=erf^{-1}(E_{x})/\sqrt{2}$ represents the preliminary solution value of the spot position. This value determines the approximate position of the spot on the photosensitive surface. It is not difficult to see from Equation (18) that the size of the incident spot radius also affects the final spot position coordinate value. We take the size of the incident spot into consideration in the error compensation item $\theta_{e}=\omega \cdot \lambda (x_{0})$, and use the error compensation term to compensate for the effect of the change in the spot size on the detection accuracy.

We built a spot position detection system based on the 4QD to move the beam at equal intervals along the *x*-axis of the detector. We recorded the actual coordinates of each position and the current value of each quadrant during the movement. Through calibration, we can obtain the $g(E_{xi})$ value of each point and the error compensation term.

Through least square fitting, we can finally determine the spot position detection result expression of the improved polynomial fitting algorithm as

(19) $$x=\frac{erf^{-1}(\sigma_{x})}{\sqrt{2}}\cdot \omega \cdot (a_{0}+\sum_{i=1}^{n}a_{i}x_{o}^{i}),$$

So far, we have developed a new spot position detection model. This model takes into account the spot size and the gap size. Compared with traditional spot position detection algorithms, this model not only effectively improves detection accuracy, but is also insensitive to changes in spot size.

## 4. Experimental Platform and Result Analysis

**Figure 6:**
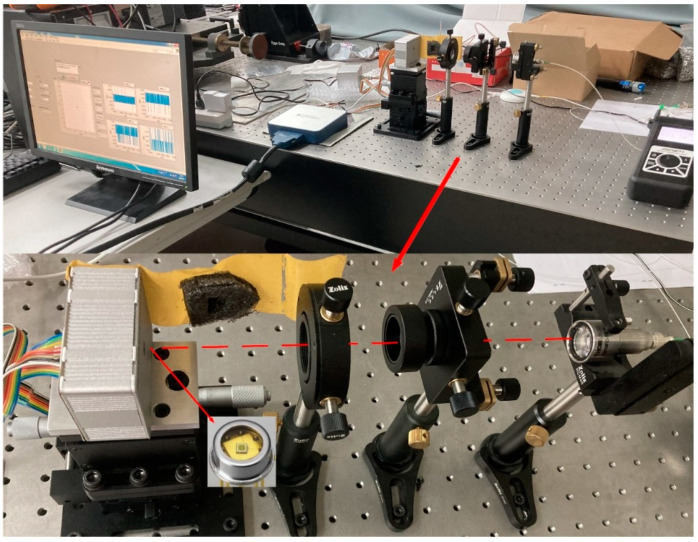
Experimental device for spot position detection.

**Figure 7:**
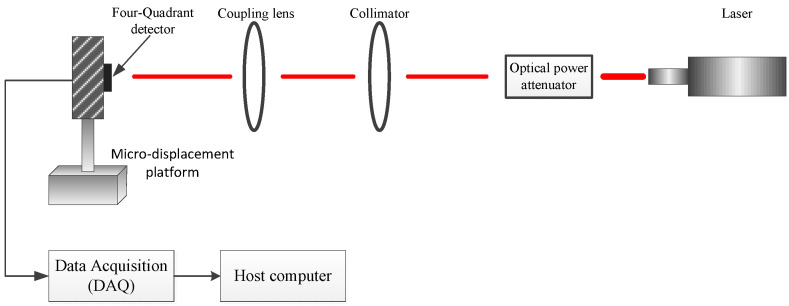
Block diagram of experimental equipment.

In order to verify the accuracy of the 4QD detection in a high-background-noise environment, we built a spot position detection system. The experiment platform is shown in Figure 6. This system consists of a laser with an emission wavelength of 1550 nm, an optical power attenuator, a collimator, a coupling lens and an InGaAs 4QD detector (HAMAMATSU G6849-01). The diameter of the photo-sensitive surface of the detector is 1 mm, the size of the gap is 0.03 mm and the photoelectric conversion efficiency is 0.95 A/W. The detector is mounted on a three-degrees-of-freedom micro-displacement platform. The minimum displacement of the platform is 10 µm and the accuracy is 10 nm. The output signal of the 4QD detector is converted and amplified from current to voltage through a transimpedance amplifier circuit. The circuit magnification is $4\times 10^{8}\;V/A$ and the maximum input current is 25 nA. The amplified photoelectric signal is A/D and is converted by a NI 6115 acquisition card. The digital denoising and spot position calculation are performed using the host computer. The block diagram of experimental equipment is shown in Figure 7. All equipment parameters and models used in this experiment are shown in Table 1.

Since the *x*- and *y*-axes are independent of each other in the calculation of the spot position, we analyzed the detection accuracy of the laser spot on the *x*-axis separately. We moved the laser spot along the *x*-axis by moving the micro-displacement platform. First, we tested the dark current noise in the dark environment and the background light noise signal when there was no laser signal input. We wrote a human-computer interaction interface in the host computer to display the spot position in real time.

**Figure 8:**
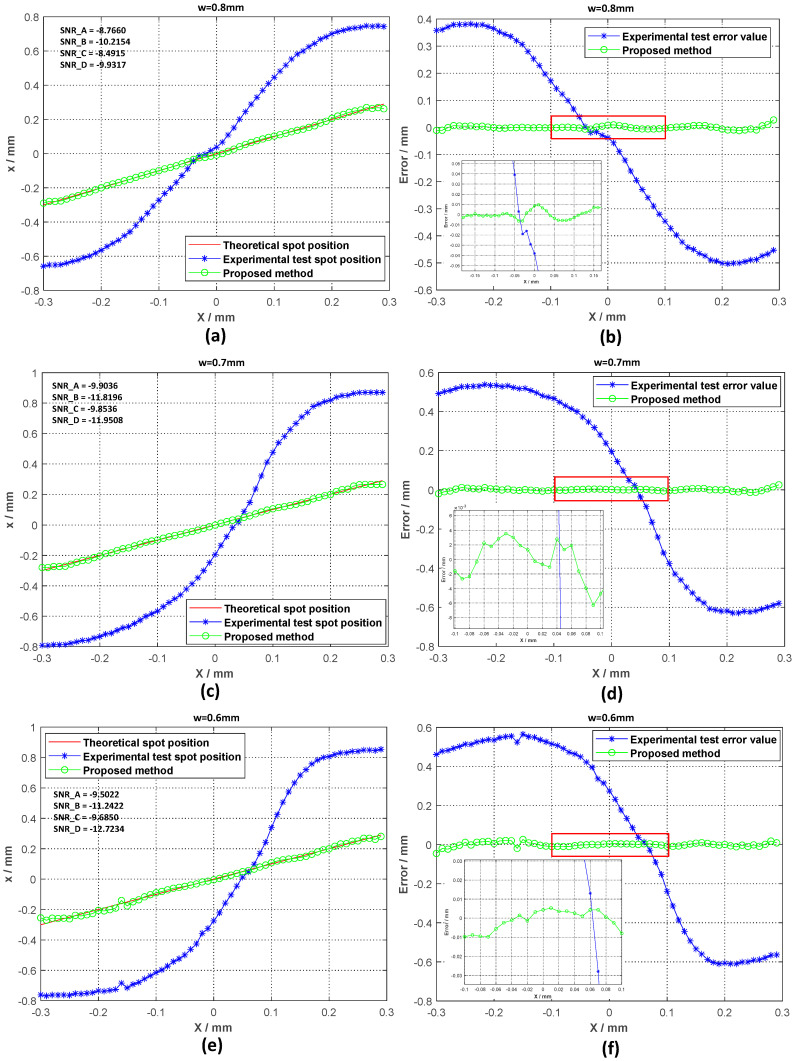
Comparison of the position detection results for three different laser spot sizes in a high-background-noise environment. (**a**) Calculation result for the *x*-axis position of the laser spot when ω = 0.8 mm; (**b**) calculation error for the *x*-axis position of the laser spot when ω = 0.8 mm; (**c**) calculation result for the *x*-axis position of the laser spot when ω = 0.7 mm; (**d**) calculation error for the *x*-axis position of the laser spot when ω = 0.7 mm; (**e**) calculation result for the *x*-axis position of the laser spot when ω = 0.6 mm; (**f**) calculation error for the *x*-axis position of the laser spot when ω = 0.6 mm.

In order to test the effectiveness of the detection method, we conducted experimental tests in a high-background-noise environment. When the radius of the incident spot was 0.6 mm, 0.7 mm, and 0.8 mm, we moved the spot along the *x*-axis and recorded the voltage data every 10 µm. Through the collected voltage, the spot position was calculated using Formula (1) and compared with the method proposed in this paper. In order to demonstrate the effectiveness of the method more intuitively, the spot position detection error is also given in Figure 8.

**Figure 9:**
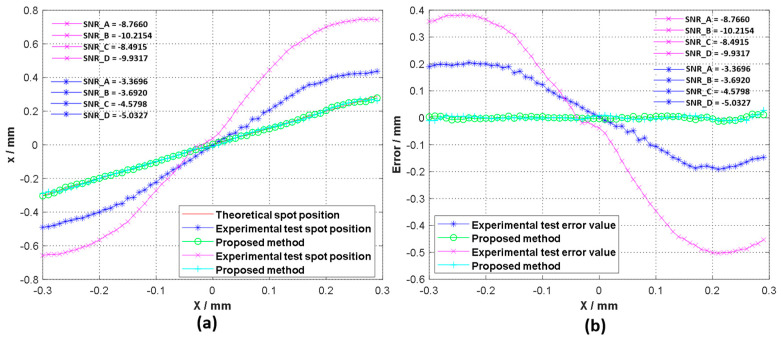
(**a**) Calculation results for the *x*-axis position of the laser spot under two different test environments when ω = 0.8; (**b**) calculation error for the *x*-axis position of the laser spot under two different test environments when ω = 0.6 mm.

The average values of the electrical SNR of each quadrant for each group of tests are shown in Figure 7. SNR_A, SNR_B, SNR_C, and SNR_D represent the output electrical SNR of the four quadrants. It can be seen that the low SNR has a greater influence on the accuracy of spot position detection. After Kalman filtering of the signal and spot position calculation, the spot position obtained is close to the theoretical value. It can be seen that the method proposed in this paper can greatly improve the spot position detection of the 4QD in a high-background-noise environment, and can maintain an improved detection accuracy in a larger range. In order to compare the influence of the SNR on the detection accuracy of the spot position, we changed the experimental environment when the size of the incident spot was 0.8 mm (that is, we changed the SNR). The test results are shown in Figure 9.

We can see from Figure 8 that a lower SNR makes the spot position detection accuracy worse, which also verifies Equation (5). Low SNR reduces the spot position detection accuracy.

**Figure 10:**
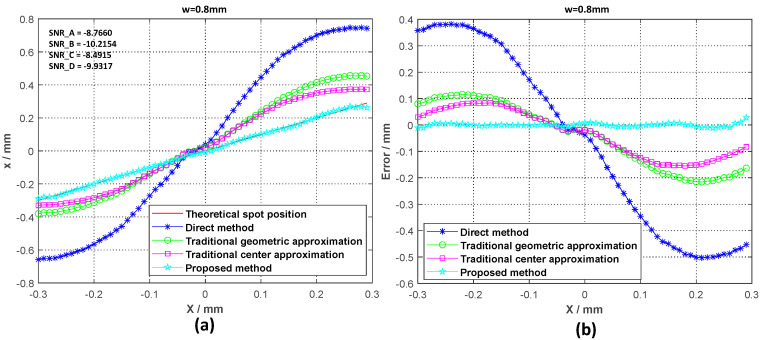
(**a**) Position calculation comparison results for the four spot position detection algorithms when ω = 0.8; (**b**) position calculation error comparison results for the four spot position detection algorithms when ω = 0.8.

In addition, we compared the traditional spot position detection algorithm with the spot position detection method proposed in this paper with the same low SNR data. We used a set of experimental data to compare the spot position detection accuracy of the four methods, which are (i) the direct calculation method of direct formula calculation with experimental observation data, (ii) the traditional center approximation method, (iii) the traditional geometric approximation method, and (iv) the method proposed in this paper. The comparison results for these four methods are shown in Figure 10.

Finally, we calculated the maximum error value $e_{MAX}$ and the root mean square error (RMSE) value $e_{RMSE}$ for the spot position detection method. The position detection results for different spot sizes in a high-background-noise environment were compared and are shown in Table 2. A comparison of the detection results for the different detection methods under the same spot size and SNR environment is shown in Table 3.

From the data in Table 2 and Table 3, it can be seen more intuitively that the spot size and the 4QD output signal SNR have an influence on the spot position detection accuracy. However, compared to the direct method of using experimental measurement data to calculate the spot position using Equation (1), the detection accuracy of our proposed method is not sensitive to changes in spot size and SNR. Even in an environment with a low SNR, the root mean square error is only 0.0065 mm. Comparing this with several traditional spot position detection methods shows that our method has high spot position detection accuracy in a high-background-noise environment. Therefore, this method is more suitable for complex application environments.

## 5. Conclusions

We have developed a 4QD spot position detection method for use in a high-background-noise environment. Using a model, we analyzed the principles of 4QD spot position detection and several factors that affect its accuracy. In a low SNR environment, the 4QD spot position detection accuracy will be significantly reduced, which affects the precision of tracking and positioning in a free space optical communication system. We therefore proposed a new spot position detection method. First, we used a transimpedance amplifier circuit to convert and amplify the 4QD output current signal from current to voltage. The signal was then collected by an A/D, transmitted to the host computer and denoised using a Kalman filter. The denoised signal was calculated using the improved spot position detection model. The improved model includes the spot size and the gap size of the 4QD photo-sensitive surface, which can further improve spot position detection accuracy. Through experimental verification, the maximum detection error obtained using the new detection method in a low SNR environment was 0.0277 mm and the root mean square error was 0.0065 mm. Furthermore, through experimental comparison, the detection accuracy of the new method was less sensitive to changes in spot size and SNR and maintained high detection accuracy in a large detection range. The new spot position detection method has many potential applications and can perform well in complex use environments.

## Figures and Tables

**Table 1:** Experimental equipment parameters and models.

Experimental Apparatus	Parameters/Model
Four-Quadrant detector	Photosensitive area φ1 mm Photo sensitivity 0.95 A/W Spectral response range 0.9–1.7 µm
Laser	Continuous wavelength 1550 nm
Collimator	THORLABS F810FC-1550
Coupling lens	Focal Length 100 mm
Optical power attenuator	Attenuable wavelength range 1260~1650 nm
Data Acquisition (DAQ)	Sampling rate 10 MS/s
Micro displacement platform	Minimum Displacement 10 µm Accuracy 10 nm

**Table 2:** Spot position detection errors under different spot sizes and signal-to-noise (SNR) ratios.

Spot Radius (mm)	SNR (dB)	Direct Method		Proposed Method	
		$e_{MAX}(mm)$	$e_{RMSE}(mm)$	$e_{MAX}(mm)$	$e_{RMSE}(mm)$
0.6	SNR_A = −9.5022 SNR_B = −11.2422 SNR_C = −9.6850 SNR_D = −12.7234	0.5650	0.4761	0.0263	0.0116
0.7	SNR_A = −9.9036 SNR_B = −11.8196 SNR_C = −9.8536 SNR_D = −11.9508	0.5380	0.4764	0.0251	0.0069
0.8	SNR_A = −8.7660 SNR_B = −10.2154 SNR_C = −8.4915 SNR_D = −9.9317	0.3820	0.3437	0.0277	0.0065
0.8	SNR_A = −3.3696 SNR_B = −3.6920 SNR_C = −4.5798 SNR_D = −5.0327	0.2050	0.1488	0.0136	0.0054

**Table 3:** Errors of four spot position detection methods.

Spot Radius (mm)	SNR (dB)	Method	$e_{MAX}(mm)$	$e_{RMSE}(mm)$
W = 0.8 mm	SNR_A = −8.7660 SNR_B = −10.2154 SNR_C = −8.4915 SNR_D = −9.9317	Direct method	0.3820	0.3437
		Traditional geometric approximation	0.1159	0.1279
		Traditional center approximation	0.0837	0.0930
		Proposed method	0.0277	0.0065
